# AaZFP3, a Novel CCCH-Type Zinc Finger Protein from *Adonis amurensis*, Promotes Early Flowering in *Arabidopsis* by Regulating the Expression of Flowering-Related Genes

**DOI:** 10.3390/ijms23158166

**Published:** 2022-07-25

**Authors:** Meiqi Wang, Haizhen Zhang, Shengyue Dai, Shuang Feng, Shufang Gong, Jingang Wang, Aimin Zhou

**Affiliations:** College of Horticulture and Landscape Architecture, Northeast Agricultural University, Harbin 150030, China; meiqiwang1998@163.com (M.W.); haizhenzhang@neau.edu.cn (H.Z.); shengyuedai@163.com (S.D.); fengshuang86@163.com (S.F.); shufanggong@neau.edu.cn (S.G.)

**Keywords:** *Adonis amurensis*, CCCH-type zinc finger protein, flowering, flowering-time genes

## Abstract

CCCH-type zinc finger proteins (ZFP) are a large family of proteins that play various important roles in plant growth and development; however, the functions of most proteins in this family are uncharacterized. In this study, a CCCH-type ZFP, *AaZFP3,* was identified in the floral organ of *Adonis amurensis*. Quantitative real-time PCR (qPCR) analysis revealed that *AaZFP3* was widely expressed in the flowers of *A.* *amurensis*. Subcellular localization analysis showed that the AaZFP3 protein was mainly localized to the cytoplasm in tobacco and *Arabidopsis*. Furthermore, the overexpression of *AaZFP3* promoted early flowering in *Arabidopsis* under both normal and relatively low-temperature conditions. RNA-sequencing and qPCR analyses revealed that the expression of multiple key flowering-time genes was altered in transgenic *Arabidopsis* overexpressing *AaZFP3* compared to wild-type. Of these genes, *FLOWERING LOCUS T* (*AtFT*) expression was most significantly up-regulated, whereas *FLOWERING LOCUS C* (*AtFLC*) was significantly down-regulated. These results suggest that the overexpression of *AaZFP3* promotes early flowering in *Arabidopsis* by affecting the expression of flowering-time genes. Overall, our study indicates that AaZFP3 may be involved in flowering regulation in *A.* *amurensis* and may represent an important genetic resource for improving flowering-time control in other ornamental plants or crops.

## 1. Introduction

Zinc finger proteins (ZFPs) are a superfamily involved in many important biological processes in plant growth, development, and stress responses [1]. The zinc finger motif is a local peptide structure stabilized by the coordination of zinc ions by cysteines and histidines; this motif is conserved across ZFPs and functions as part of the DNA or RNA binding domain or protein–protein interaction domain [2]. CCCH-type ZFPs (also known as C3H-type ZFPs) are a group of ZFPs that usually contain one to six tandemly arranged CCCH-type zinc finger motifs comprising three cysteine (“CCC”) residues and one subsequent histidine (“H”) residue [3,4]. A consensus sequence for CCCH-type motifs was defined as CX_4-17_-CX_4-6_-CX_3_-H (where X represents any amino acid) based on whole-genome analysis of CCCH-type ZFP proteins in *Arabidopsis thaliana* and *Oryza sativa* [4]. To date, 68, 67, 68, 69, and 53 CCCH-type ZFP proteins have been identified in *Arabidopsis*, *O. sativa*, *Zea mays*, *Vitis vinifera*, and *Hordeum vulgare*, respectively [4,5,6,7]. However, the characterization of their functions is very limited.

In the model plant *Arabidopsis*, the functions of several members of the CCCH-type ZFP family have been reported [8]. For example, *Arabidopsis* AtHUA1, a CCCH-type ZFP with six tandem zinc-finger (TZF) motifs, has been identified as an RNA-binding protein that may be involved in flower development [9]. The CCCH-type ZFP AtC3H23/AtTZF1 traffics between the nucleus and cytoplasmic foci and can bind both DNA and RNA and is likely involved in gibberellin/abscisic acid-mediated developmental and environmental responses [10]. AtC3H20/AtTZF2 and AtC3H49/AtTZF3 were reported to be involved in abscisic acid and jasmonic acid responses by affecting mRNA turnover processes [11]. AtC3H14 and AtC3H15 play roles in the regulation of secondary wall thickening and anther development by participating in both transcriptional and post-transcriptional processes [12]. AtC3H36/AtKHZ1 [CCCH zinc-finger and K homology (KH) domain proteins] and AtC3H52/AtKHZ2 both possess transactivation activities and RNA-binding abilities and have redundant roles in the regulation of flowering and senescence in *Arabidopsis* [13]. AtC3H17, a non-tandem CCCH-type ZFP, functions as a nuclear transcriptional activator and has pleiotropic effects on vegetative, flowering, seed development, and salt stress responses [14,15]. Taken together, these studies indicate the importance of CCCH-type ZFPs for a variety of processes in *Arabidopsis*; however, the functions of most members of this ZFP family remain uncharacterized.

In addition, the functions of only a few members of the CCCH-type ZFP family have been studied in other plants. For example, the *O. sativa* CCCH-type ZFP, OsDOS, was reported to be involved in delaying leaf senescence [16]. GhZFP1 from *Gossypium hirsutum* was found to enhance salt stress tolerance and fungal disease resistance in transgenic tobacco [17]. IbC3H18 from *Ipomoea batatas* functions as a nuclear transcriptional activator and enhances abiotic stress tolerance [18]. These studies suggest broad roles for CCCH-type ZFPs in plant growth and development.

In both *Arabidopsis* and *V. vinifera*, CCCH-type ZFP3 is a member of a ZFP family that contains five TZF motifs [4,5]; however, regarding its function, it is completely unknown. In our previous study, a CCCH-type ZFP member, *AaZFP3*, was identified from the floral organ of *Adonis amurensis* [19], suggesting that it may play an important role in flowering regulation. *A. amurensis* is a perennial herbaceous flower in the family Ranunculaceae, which is naturally distributed in northeast China. *A. amurensis* can blossom naturally under extremely low temperatures (day/night temperatures between −15 and 10 °C) [19]. Therefore, it is the ideal plant species to study flowering control at extremely low temperatures. In this study, the function of *AaZFP3* in flowering regulation was further investigated in *Arabidopsis* through transgenic experiments. Our results revealed that overexpression of *AaZFP3* promotes early flowering in *Arabidopsis* under both normal (22 °C) and relatively low temperature (18 °C) conditions, which may be related to the altered expression of flowering time-related genes.

## 2. Results

### 2.1. Sequence and Expression Analysis of AaZFP3

**Figure 1:**
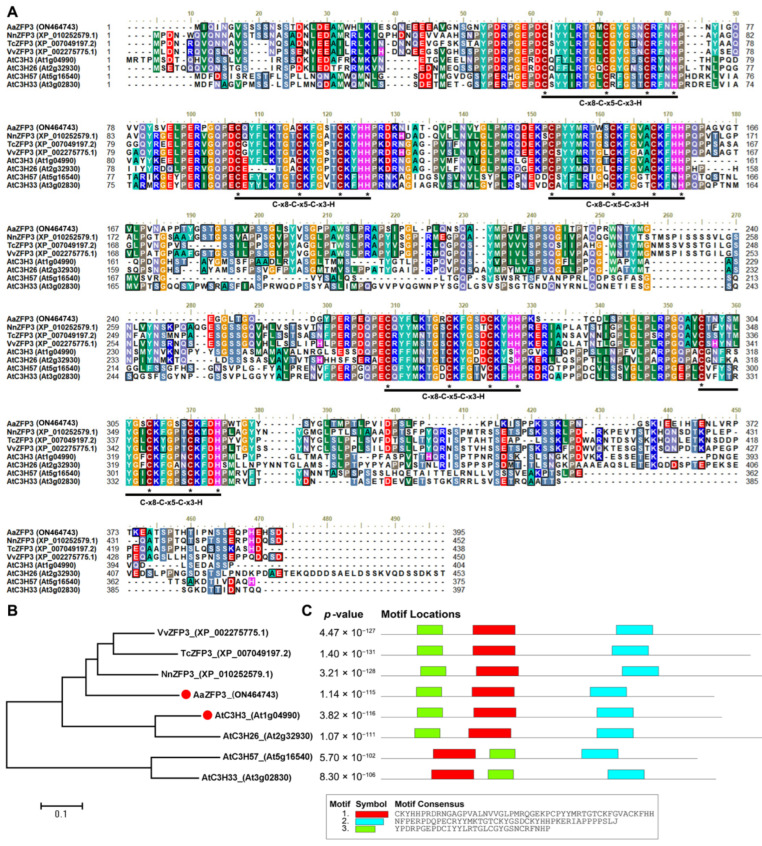
Sequence analysis of AaZFP3. (**A**) Multiple sequence alignment of AaZFP3 with other zinc finger proteins from multiple species, including *Arabidopsis thaliana*, *Nelumbo nucifera*, *Vitis vinifera*, and *Theobroma cacao*. The *Arabidopsis* ZFP protein is also known as C3H. Asterisks indicate the conserved cysteine (C) and histidine (H) residues. (**B**) Phylogenetic tree of ZFP3 proteins. (**C**) Conserved motifs of ZFP3 proteins.

**Figure 2:**
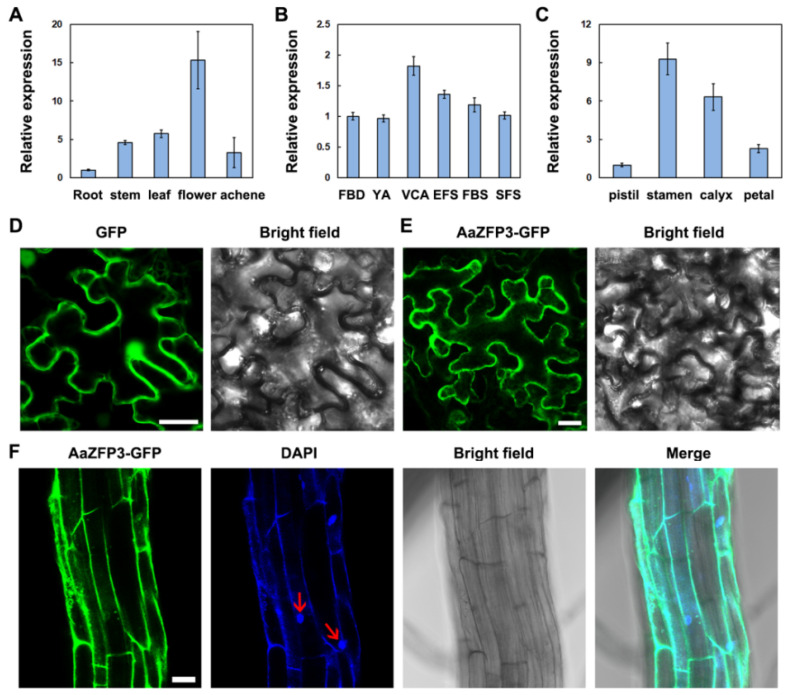
Tissue expression specificity and subcellular localization of *AaZFP3*. (**A**–**C**) Relative expression levels of the *AaZFP3* gene in (**A**) various organs, (**B**) floral organs at different developmental stages, and (**C**) different flower tissues in *Adonis amurensis.* FBD, flower bud differentiation; YA, young alabastrum; VCA, visible color alabastrum; EFS, early flowering stage; FBS, full bloom stage; SFS, senescing flower stage. The *AaActin* gene provided the internal control, and the transcript level in the root or floral organs at flower bud differentiation or pistil was consistently 1.0, respectively. (**D**,**E**) Confocal images of tobacco leaf cells transiently expressing (**D**) GFP (control) or (**E**) AaZFP3-GFP. (**F**) Confocal images of transgenic *Arabidopsis* stem cells stably expressing AaZFP3-GFP (green) with DAPI (blue) staining. Red arrows indicate nuclei stained with DAPI. Scale bars = 20 μm.

The full-length open reading frame (ORF) of *AaZFP3* consisted of 1188 bp, encoding 395 amino acids. Sequence analysis showed that *AaZFP3* contains five conserved C-x8-C-x5-C-x3-H motifs (Figure 1A). The phylogenetic and conserved motif analyses revealed that AaZFP3 was more similar to *NnZFP3* in *Nelumbo nucifera* (Figure 1B,C). In the *Arabidopsis* CCCH-type ZFP family, it is most similar to AtC3H3 (Figure 1B,C). In terms of its expression profile, *AaZFP3* exhibited the highest expression in *A.* *amurensis* flowers, followed by leaves and stems, with the lowest expression in roots (Figure 2A). During the six stages of flower development, *AaZFP3* expression was higher in flowers at the visible color alabastrum (VCA) and the early flowering stage (EFS) (Figure 2B). Moreover, *AaZFP3* expression was higher in the stamens and calyx than in the petals and pistil in the floral organs of *A.* *amurensis* (Figure 2C). The subcellular localization of AaZFP3 was investigated using transient and stable expression methods involving green fluorescent protein (GFP) fusion. In tobacco leaf epidermal cells, the AaZFP3-GFP fluorescence signal was similar to the control GFP fluorescence signal (Figure 2D,E). In the stem cells of transgenic *Arabidopsis*, the AaZFP3-GFP signal was mainly distributed in the cytoplasm and did not appear to overlap with the nuclear dye DAPI (Figure 2F). These results suggest that AaZFP3 is primarily localized in the cytoplasm.

### 2.2. Effect of AaZFP3 Overexpression on Flowering Time in Arabidopsis

**Figure 3:**
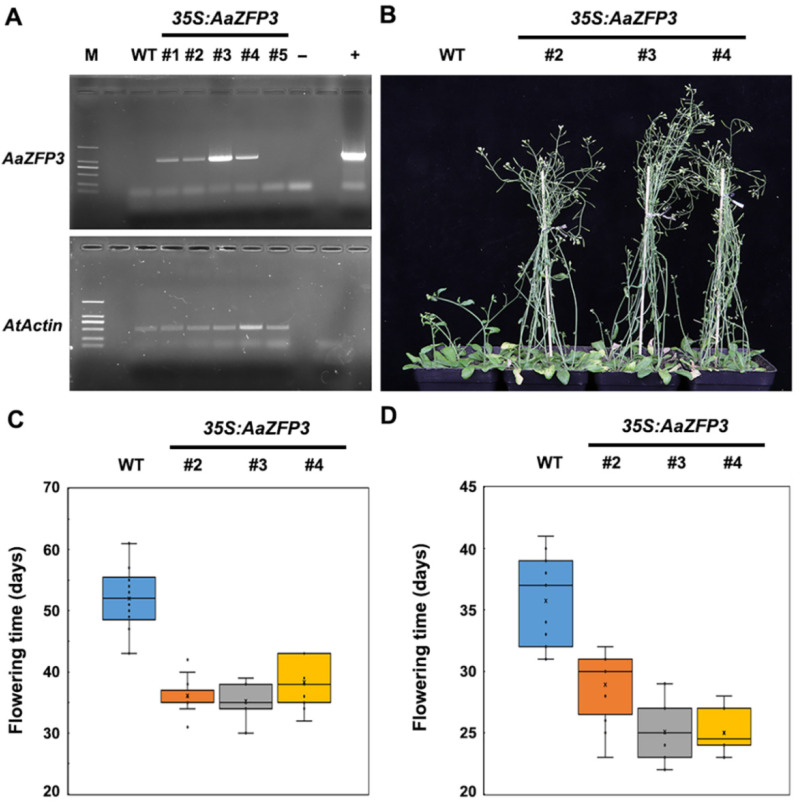
Flowering time of soil-grown wild-type (WT) plants and transgenic *Arabidopsis* plants overexpressing *AaZFP3*. (**A**) Reverse transcript-PCR analysis of *AaZFP3* expression in WT and transgenic *Arabidopsis* lines (#1~#5). *AtActin1* (At2g37620) was used as an internal control. M, marker; +, positive control; −, negative control (H_2_O). (**B**) Flowering phenotype of WT and transgenic *Arabidopsis* plants (#2, #3, #4) grown in soil under a 12 light/12 h dark photoperiod at 22 °C. (**C**,**D**) Box plots of flowering time for WT and transgenic *Arabidopsis* plants (#2, #3, #4) under a 12 light/12 h dark (**C**) and 16 light/8 h dark (**D**) photoperiod at 22 °C. The interquartile range box represents the middle 50% of the data. Whiskers extend 1.5 times the interquartile range and the center line marks the median. *n* ≥ 15 plants per line.

**Figure 4:**
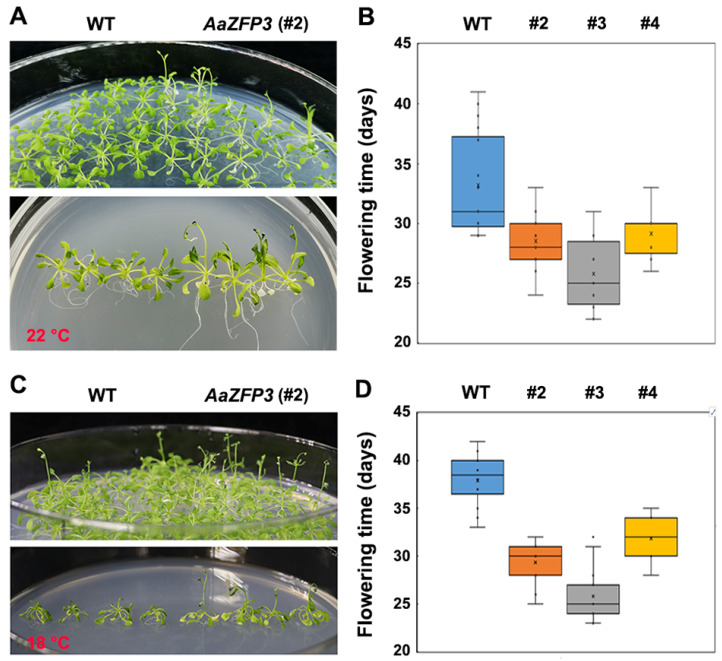
Flowering time of medium-grown wild-type (WT) and transgenic *Arabidopsis* plants overexpressing *AaZFP3*. Flowering phenotype and box plots of flowering time of WT and transgenic *Arabidopsis* plants (#2, #3, #4) grown on plates containing 1/2 MS medium under a 12 light/12 h dark photoperiod at 22 °C (**A**,**C**) or 18 °C (**B**,**D**). The interquartile range box represents the middle 50% of the data. Whiskers extend 1.5 times the interquartile range and the center line marks the median. *n* ≥ 15 plants pre line.

To investigate the function of *AaZFP3*, we generated transgenic *Arabidopsis* overexpressing *AaZFP3* driven by the 35 S promoter (Figure 3A). Soil-grown, mature transgenic *Arabidopsis* plants displayed an early flowering phenotype compared to wild-type (WT) plants under a 12 h light/12h dark photoperiod at 22 °C (Figure 3B,C). Under a 16 h light/8 h dark photoperiod, transgenic *Arabidopsis* plants also have shorter flowering times than WT plants (Figure 3D). Furthermore, the early flowering phenotype was also observed in transgenic *Arabidopsis* plants grown on 1/2 strength Murashige and Skoog (MS) plates under a 12 h light/12 h dark photoperiod at 22 °C (Figure 4A,B). At relatively low temperatures (18 °C) conditions, transgenic *Arabidopsis* plants grown on 1/2 strength MS plates also have shorter flowering times than WT plants (Figure 4C,D). These results suggest that the overexpression of *AaZFP3* causes early flowering in *Arabidopsis* under normal and relatively low-temperature conditions.

### 2.3. Effect of ZFP3 Overexpression on the Expression of Flowering-Related Genes in Arabidopsis

**Figure 5:**
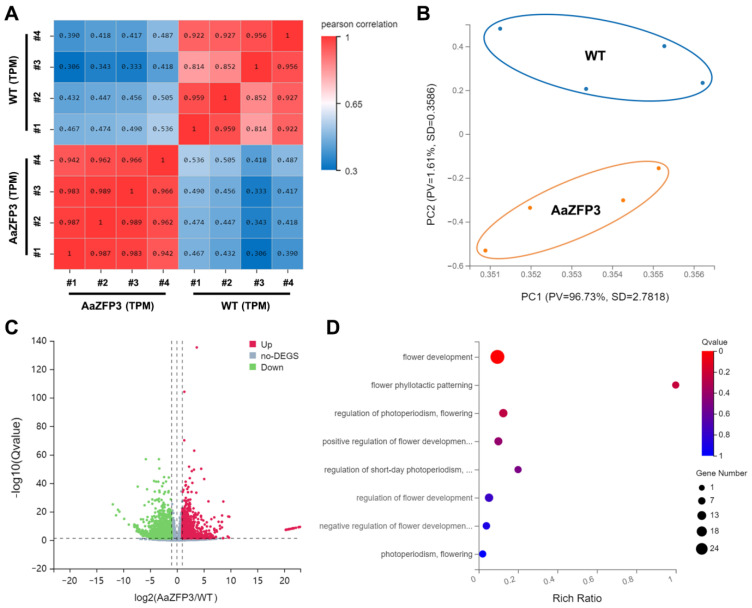
RNA-sequencing analysis of differentially expressed genes (DEGs) between wild-type (WT) and transgenic *Arabidopsis* plants overexpressing *AaZFP3*. (**A**) Correlation matrix heatmap showing pairwise Pearson correlation coefficients, represented by the color scale. (**B**) Principal component analysis (PCA) of gene expression from eight RNA-seq samples. (**C**) Volcano plot showing the number of DEGs in *AaZFP3*-overexpressing transgenic versus WT *Arabidopsis* plants. The abundance of each gene was normalized as transcripts per million (TPM). We used a false discovery rate ≤ 0.05 and an absolute value of log2Ratio ≥ 2 as the threshold for significance. Red, up-regulated; blue, down-regulated; gray, no significant change (no-DEGs). (**D**) GO term enrichment analysis of all DEGs involved in flowering regulation-related GO terms. The rich ratio is the ratio of the number of DEGs annotated under a given GO term to the total number of genes annotated under the GO term. The *q*-value is the corrected *p*-value and ranges from 0 to 1 as represented by the color scale. The size of the dot indicates the number of genes contributing to the GO term.

**Figure 6:**
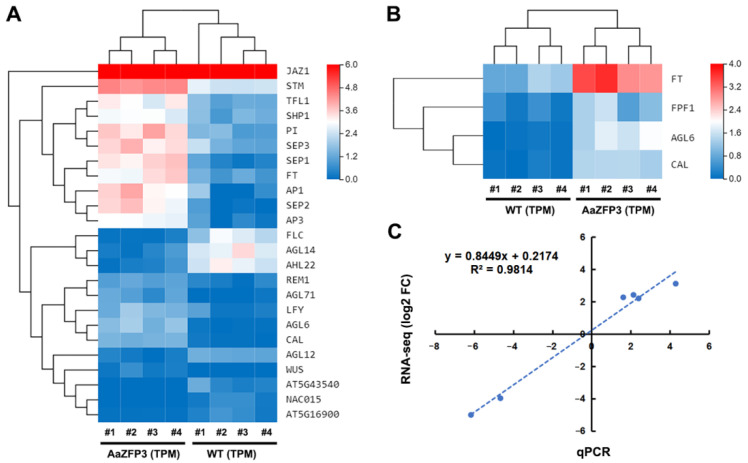
The RNA-seq expression levels of differentially expressed genes (DEGs) categorized in the (**A**) “flower development” and (**B**) “positive regulation of flower development” GO terms in WT and transgenic *Arabidopsis* plants. Red, up-regulated genes; green, down-regulated genes. (**C**) Correlation analysis between the qPCR and RNA-seq data. Detailed information on the gene names is provided in Supplementary Table S1.

**Figure 7:**
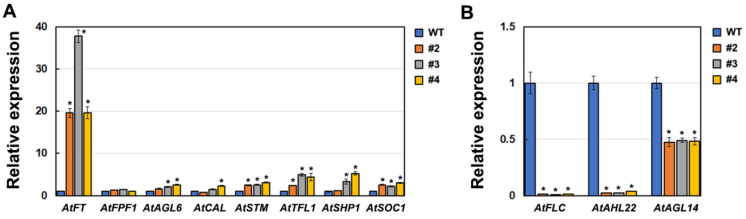
qPCR validation of key differentially expressed genes (DEGs) involved in flowering time control and flower development. (**A**,**B**) qPCR results showing the relative expression levels of (**A**) up-regulated and (**B**) down-regulated DEGs in transgenic *Arabidopsis* lines compared to WT plants. The *AtActin* gene was used as an internal control and its transcript level in WT plants was set to 1.0. Error bars represent the standard error (*n* = 3). Asterisks indicate significant differences between the transgenic lines and WT plants (Student’s *t*-test; *p* < 0.01). Detailed information on the gene names is provided in Supplementary Table S1.

RNA-sequencing (RNA-seq) was performed to identify differentially expressed genes (DEGs) between WT and transgenic *Arabidopsis* plants. Four samples from each condition were sequenced. The correlation matrix and principal component analysis based on gene expression demonstrated that the samples clustered by condition, as expected (Figure 5A,B). DEGs were screened using the transcripts per million (TPM) method with a false discovery rate (FDR) threshold of 0.05 and an absolute log2 ratio of 2. In total, we identified 1364 DEGs, of which 903 were down-regulated and 461 were up-regulated in transgenic *Arabidopsis* compared to WT (Figure 5C). Gene Ontology (GO) enrichment analysis of these DEGs identified eight over-represented flower-development-related GO terms, which contained a total of 25 genes related to flowering or flower development (Figure 5D; Table S1). Furthermore, the majority of DEGs included under the “flower development” or “positive regulation of flower development” GO terms exhibited differential expression profiles in WT versus transgenic *Arabidopsis* plants (Figure 6A,B). Under the “positive regulation of flower development” GO term, four DEGs were identified, including *FLOWERING LOCUS T* (*FT*), *flowering promoting factor 1* (*FPK1*), *AGAMOUS-LIKE6* (*AGL6*), and *CAULIFLOWER* (*CAL*) (Figure 6B). Quantitative real-time PCR (qPCR) confirmed the reliability of the RNA-seq data (Figure 6C). Interestingly, both RNA-seq and qPCR detected significantly higher (20 folds) expression of *FT* in transgenic *Arabidopsis* plants than in WT plants, and the fold change of *FT* expression was significantly higher than that of other DEGs (Figure 7A). We also detected up-regulation (2.5 folds) of *SUPPRESSOR OF OVEREXPRESSION OF CONSTANS1* (*AtSOC1*) (Figure 7A), an integrator of the *Arabidopsis* flowering pathway. In addition, significantly lower expression of two flowering suppression-related genes, *FLOWERING LOCUS T* (*FLC*) and *AT-hook motif nuclear localized 22* (*AHL22*), was observed in transgenic *Arabidopsis* plants compared to WT plants (Figure 7B). These results suggest that the overexpression of *AaZFP3* affects the expression of flowering-related genes, which may contribute to the early flowering phenotype observed in *AaZFP3-*overexpressing *Arabidopsis*.

**Figure 8:**
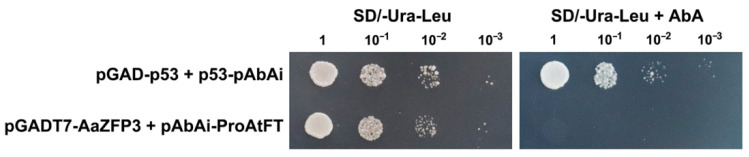
Yeast one hybrid (Y1H) assay of AaZFP3 binding to *AtFT* promoter. The full lengths of *AaZFP3* and *AtFT* promoter (1907 bp) were constructed pGADT7 and pAbAi vectors, respectively, and they were subsequently cotransformed with yeast strain Y1HGold. Transformed yeast was grown in SD/-Ura-Leu media with or without AbA (0.6 mg L^−1^). The pGAD-p53 + p53-pAbAi was used as a positive control. Pro: promoter.

In addition, whether AaZFP3 binds to the promoter (Pro) of *AtFT* was investigated using yeast one hybrid (Y1H). Y1H assays showed that when fused pAbAi-ProAtFT was coexpressed with pGADT7-AaZFP3 in yeast, the yeast strains could not grow on SD/-Ura/-Leu plates with AbA (Figure 8). This result suggests that AaZFP3 does not activate the promoter of the *AtFT* gene, and that it may affect *AtFT* expression in other ways.

## 3. Discussion

Several studies have demonstrated important roles for CCCH-type ZFPs in flowering regulation. For example, the overexpression of *AtC3H17*, *AtC3H36/AtKHZ1,* and *AtC3H52/AtKHZ2* promoted early flowering in *Arabidopsis* [13,14], while the overexpression of *AtC3H23/AtTZF1* delayed flowering [20,21]. Notably, AtC3H17, AtC3H36/AtKHZ1, and AtC3H52/AtKHZ2 were found to be specifically localized to the nucleus, whereas AtC3H23/AtTZF1 was found to traffic between the nucleus and cytoplasmic foci [10,13,14]. The localization of CCCH-type ZFPs in the nucleus or cytoplasm may be related to their propensity to bind DNA or RNA. These studies suggest that different members of the CCCH-type ZFP family may regulate flowering regulation via different mechanisms, including transcriptional and post-transcriptional regulation through DNA or RNA binding.

**Figure 9:**
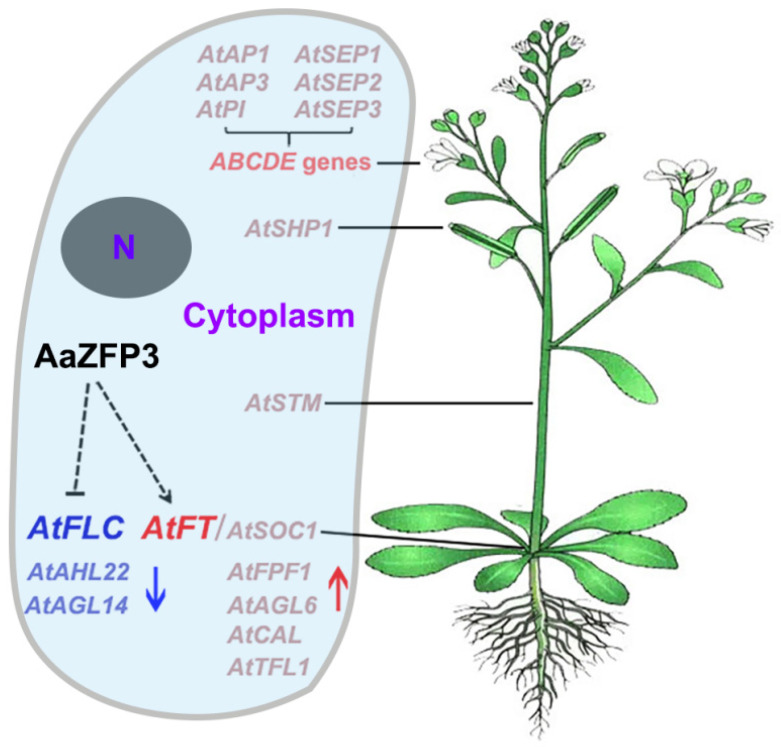
Proposed model of the genetic regulation of *Arabidopsis* flowering by *AaZFP3*. Red (upward arrow), genes up-regulated following *AaZFP3* overexpression; blue (downward arrow), genes down-regulated following *AaZFP3* overexpression; N, nucleus. Detailed information on the gene names is provided in Supplementary Table S1.

*AtFLC* and *AtFT* are two key genes involved in flowering regulation in *Arabidopsis* [22]. *AtFLC* directly represses *AtFT* expression during vegetative phases to inhibit the transition to flowering [23,24], and *AtFT* represses *AtFLC* expression in seedlings and leaves to promote flowering [25,26]. In our study, the overexpression of *AaZFP3* promoted early flowering in *Arabidopsis,* and the expression of *AtFT* in transgenic *Arabidopsis* plants was significantly higher than in WT plants, whereas *AtFLC* expression was significantly lower. Thus, we propose that the early flowering phenotype caused by *AaZFP3* overexpression may be driven by the increased *AtFT* expression and/or decreased *AtFLC* expression (Figure 9). This is similar to previous studies that showed that the overexpression of two CCCH-type ZFPs, *AtTZF1* and *MsZFN*, from *Arabidopsis* and *Medicago sativa*, respectively, delayed flowering in transgenic *Arabidopsis* by enhancing *AtFLC* expression and decreasing *AtFT* expression [20,27]. Furthermore, recent reports indicate that AtC3H36/AtKHZ1 and AtC3H52/AtKHZ2 can promote flowering by repressing the splicing efficiency of *AtFLC* pre-mRNA [28]. These findings suggest that some members of CCCH-type ZFPs are involved in flowering regulation by altering flowering-time gene expression via DNA or RNA binding.

In addition to *AtFT*, *AtFLC* suppresses *AtSOC1* expression by directly binding to its promoter [29]. In contrast to the significant up-regulation of *AtFT, AtSOC1* expression was only weakly up-regulated in transgenic *Arabidopsis* overexpressing *AaZFP3*. Furthermore, the repressor of *AtFT* expression, *AtAHL22* [30], was significantly suppressed in transgenic *Arabidopsis* (Figure 7B). Taken together, these results suggest that the overexpression of *AaZFP3* likely promotes early flowering in *Arabidopsis* primarily by enhancing *AtFT* expression rather than by inhibiting *AtFLC* expression. Enhanced expression of *AtFT* may further affect the expression of genes involved in flower development regulation, such as *SHOOT MERISTEMLESS* (*AtSTM*; regulates stem meristem development [31]), *SHATTERPROOF* (*AtSHP*; controls fruit development and seed dispersal in *Arabidopsis* [32,33]), and the ABCDE model genes (*APETALA1* (*AtAP1*), *AtAP3*, *PISTILLATA* (*AtPI*), *SEPALLATA1* (*AtSEP1*), *AtSEP2*, and *AtSEP3*; control the development of *Arabidopsis* floral organs [34]) (Figure 9).

In our study, AaZFP3 was mainly localized to the cytoplasm in tobacco and *Arabidopsis* by transient and stable expression experiments (Figure 2E,F). This result is highly similar to the localization of AtC3H49/AtTZF3 and AtC3H20/AtTZF2 in tobacco [11]. AtC3H49/AtTZF3 and AtC3H20/AtTZF2 contain two TZF motifs, and their recombinant proteins displayed Rnase activity in vitro, suggesting that they may be involved in the mRNA turnover process [11]. Furthermore, the Y1H assays showed that AaZFP3 does not activate the promoter of *AtFT* (Figure 8). Together with our results, these findings suggest that AaZFP3 is most likely an RNA-binding protein localized in the cytoplasm. However, whether AaZFP3 binds to the pre-mRNA or mRNA of *AtFT* requires further investigation.

## 4. Materials and Methods

### 4.1. Plant Materials

*Adonis amurensis* plants were grown in an open field at Northeast Agricultural University (Harbin, China; 128.4° E, 45.0° N). Various organs and floral tissues of *A. amurensis* were simultaneously sampled for RNA extraction.

### 4.2. Quantitative Real-Time PCR Analysis

Total RNA extraction of the plant samples was performed using TRIzol (9108, TaKaRa, Kusatsu, Japan), and reverse transcription was performed using the PrimeScript RT Kit with gDNA Eraser (RR047A, TaKaRa). qPCR was performed using a CFX96 real-time PCR detection system (Bio-Rad, Hercules, CA, USA) and SYBR Green PCR Master Mix (RR420A, TaKaRa) according to the manufacturer’s instructions. *AaActin* or *AtActin2* (At3g18780) was used as a reference gene. Three biological and three technical replicates were performed for each sample. The primers used in this study are listed in Supplementary Table S2.

### 4.3. Gene Cloning, Vector Construction, and Plant Transformation

The ORF of *AaZFP3* (GenBank: ON464743) was amplified by PCR and cloned into pBI121 or pBI121-GFP vectors using *Xba*I and *Sac*I or *Xba*I and *Kpn*I sites, respectively (without the stop codon). The primers used in this study are listed in Supplementary Table S2. The above constructs were confirmed by sequencing and transformed into *Agrobacterium tumefaciens* strain EHA105 for plant transformation. Transient expression of the pBI121-AaZFP3-GFP construct in tobacco (*Nicotiana benthamiana*) leaves was performed as previously described [35]. *Arabidopsis* (Columbia ecotype) transformation of the pBI121-AaZFP3-GFP and pBI121-AaZFP3 constructs was performed using the floral dip method [36]. Transgenic *Arabidopsis* plants were selected on 1/2 Murashige and Skoog (MS) medium containing 30 µg mL^−^^1^ of kanamycin. The expression of *AaZFP3* in transgenic *Arabidopsis* plants was assessed by semi-quantitative reverse transcript PCR analyses. T3-generation *Arabidopsis* seeds were treated at 4 °C for 2 days and then grown on ½ MS medium or soil under a 12 h light/12 h dark photoperiod at 22 or 18 °C for phenotypic analyses. The light intensity of the growth chambers was 150 µE m^−^^2^s^−^^1^.

### 4.4. Subcellular Localization

The tobacco leaf epidermis and *Arabidopsis* stems were visualized using confocal laser scanning microscopy (CLSM; Leica TCS-SP8, Wetzlar, Germany). GFP signals were detected using 500–530 nm emission filters. *Arabidopsis* stems were incubated in 1 mL of liquid 1/2 MS medium containing 10 µM 4’,6-diamidino-2-phenylindole (DAPI; Invitrogen, CA, USA) for 30 min at room temperature. GFP and DAPI signals were detected under 500–530 nm and 420–480 nm emission filters, respectively.

### 4.5. RNA-Sequencing and Differential Gene Expression Analysis

Total RNA was extracted from both wild-type (WT) and transgenic *Arabidopsis* flowering plants grown on the 1/2 MS medium using TRIzol reagent (9108, TaKaRa). Four plants were used as biological replicates for each condition. Sequencing was conducted using an Illumina-Seq system using the BGISEQ-500 system (Beijing Genomic Institute, Shenzhen, China). RNA-seq data processing and DEG analysis were conducted as previously described [19]. DEGs were screened using the TPM method with an FDR threshold of 0.05 and an absolute log_2_ ratio of 2. Pearson correlation coefficients were calculated between two samples using the cor function in R software. Principle components analysis (PCA) was performed using the princomp function in R software. All DEGs were mapped in GO biological process terms, and significantly over-represented terms were identified based on a corrected *p* ≤ 0.05. GO enrichment analysis of the DEGs was performed by the GOseq R packages based on Wallenius non-central hyper-geometric distribution as previously described [37].

### 4.6. Y1H Assays

The promoter of *AtFT* (1907 bp) was PCR amplified and inserted into a pAbAi-BR yeast integrating vector (bait-reporter; Clontech, Palo Alto, Santa Clara, CA, USA) and the *AaZFP3* gene was cloned into the pGADT7-rec vector (Clontech). The pAbAi-ProAtFT and pGADT7-AaZFP3 vectors were cotransformed into the yeast Y1HGold strain using a Matchmaker One-Hybrid Library Construction and Screening Kit (Clontech). Cotransformed yeast cells were selected on SD/-Ura-Leu (a synthetic dropout medium lacking Ura and Leu) with or without AbA (0.6 mg L^−1^; Sigma-Aldrich, St. Louis, MO, USA), and were then incubated for 3–5 days at 30 °C. The positive control (pGAD-p53 + p53-pAbAi) was carried out in the same manner. The primers used for these analyses are listed in Supplementary Table S2.

## 5. Conclusions

In summary, we identified a CCCH-type ZFP gene, *AaZFP3*, from the floral organs of *A.* *amurensis*. AaZFP3 was mainly localized to the cytoplasm. Overexpression of *AaZFP3* promoted early flowering in *Arabidopsis*, which may be caused by the altered expression of flowering-time genes, especially *AtFT*. Our results suggest that AaZFP3 may play an important role in regulating flowering in *A.* *amurensis*. Furthermore, this gene may represent an important genetic resource for changing the flowering time of other ornamental plants or crops.

## Supplementary Materials

The following are available online at https://www.mdpi.com/article/10.3390/ijms23158166/s1.
